# Supplementary figures and images for: Comprehensive identification and expression analysis of FAR1/FHY3 genes under drought stress in maize (Zea mays L.)
Source: PeerJ. 2024 Jun 28;12:e17684. doi: 10.7717/peerj.17684 (PMC11216215; doi:10.7717/peerj.17684)

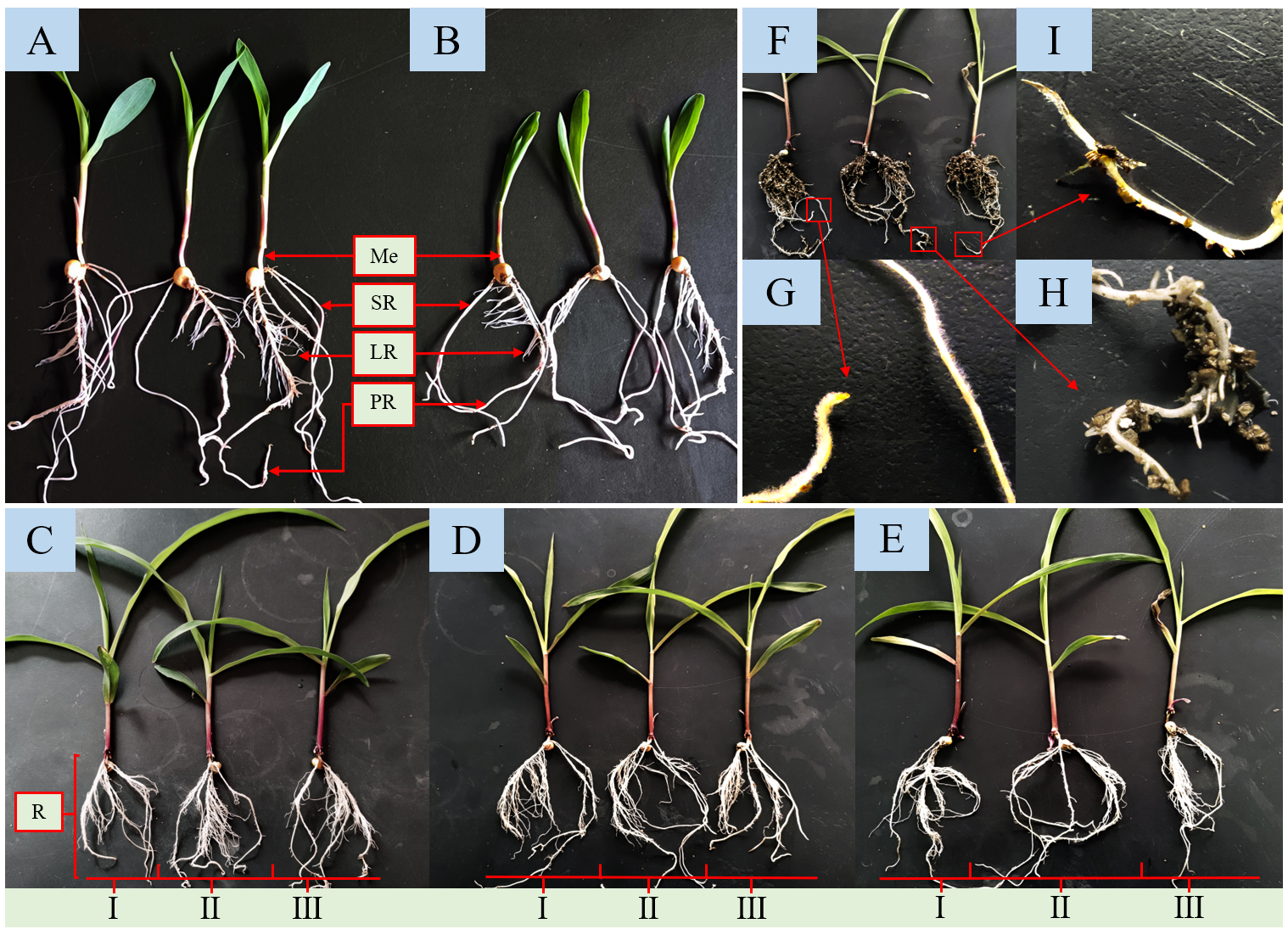

Supplement: Supplemental Information 1 — Fig. S1: The morphology of maize seedlings before and after drought treatment. (A) and (B) represents the maize seedlings at the early seedling stage with normal growth and drought treatment, respectively. The red arrow indicates the site of the root taking. ME represents mesocotyl, SR represents seminal root, LR represents lateral root, and PR represents primary root. (C), (D), and (E) represents the morphology of maize seedlings at the three-leaf stage treated for 6, 24 and 48 h, respectively. R represents roots. I, II and III represents control, 10% PEG-6000 and 25% PEG-6000 treated maize seedlings, respectively. (F) represents morphology of maize seedlings after 48 h of PEG-6000 treatment. (G), (H) and (I) represents primary root tip enlargement after 48 h of treatment with 0, 10%, and 25% PEG-6000, respectively. Fig. S2: Amino acid sequences alignment of ZmFARs using MegAlin. indicates the comparison of 23 proteins (except ZmFAR01), and (B) indicates the comparison of the all 24 ZmFARs. N-terminal WRKY-GCM1 zinc finger domain with the conserved CCHH amino acids, a central putative core transposase domain with the conserved DDG amino acids, and C-terminal SWIM zinc-finger domain of a CxCxnCXH motif are indicated, respectively. Black triangles represent conserved amino acids and red triangles represent amino acids not conserved at the site. Fig. S3: Synteny analysis of ZmFARs in maize genome. The collinearity relationship between different gene pairs was performed using MCScanX. Finally, the results were visualized using the the Dual Systeny Plot for MCScanX package in the TBtools. Fig. S4: The collinearity analysis of the ZmFAR family members between zea mays and four dicots, including Arabidopsis thaliana, Solanum tuberosum, Solanum lycopersicum, and Glycine max. Gray lines represent the collinear blocks between two plants in their genome. The MCScanX program was used to determine the covariance between different gene pairs and the results were visualized using [file peerj-12-17684-s001.zip › Supplemental materials/Supplemental figures/Fig. S1 The morphology of maize seedlings before and after drought treatment..png]

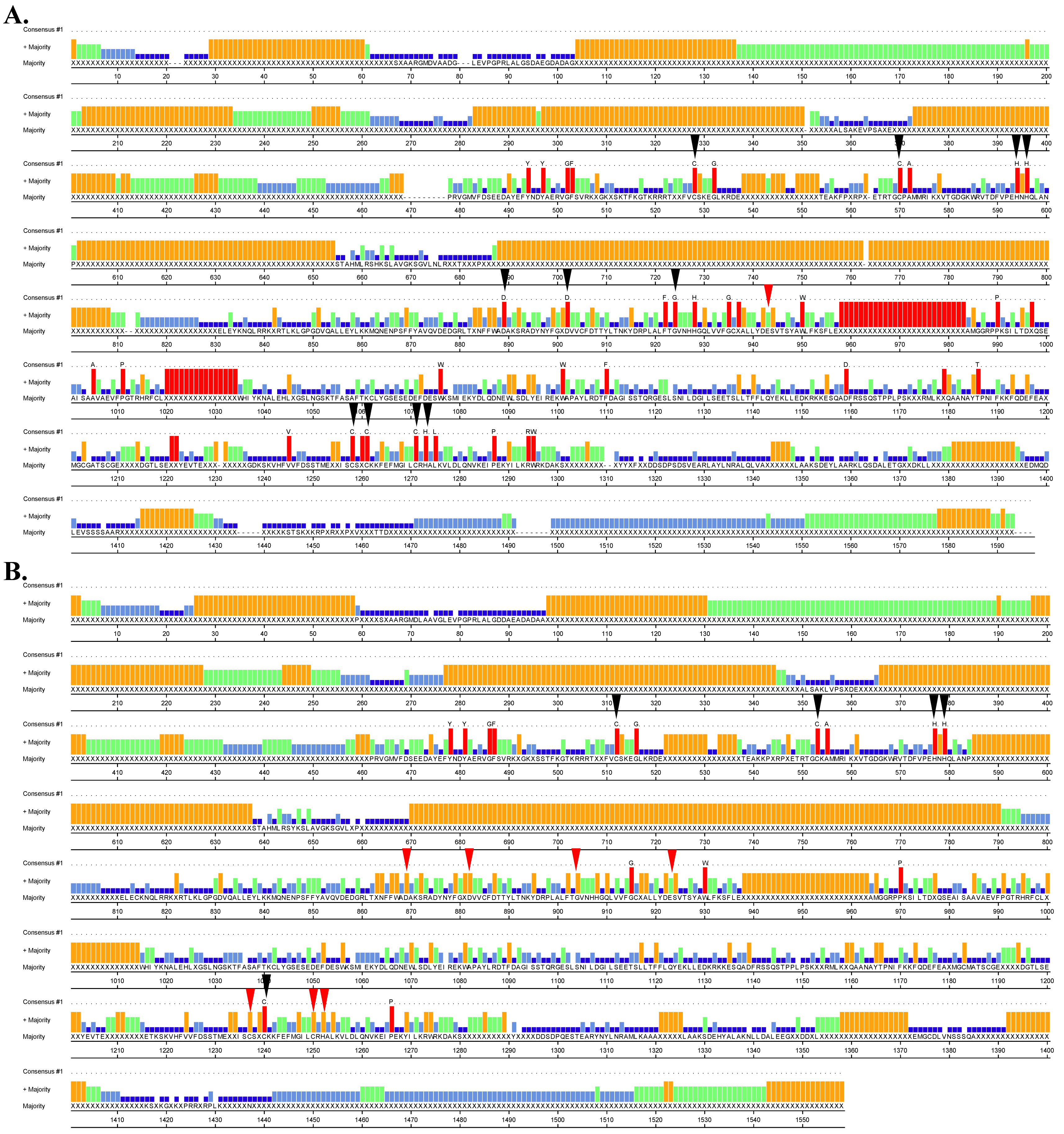

Supplement: Supplemental Information 1 — Fig. S1: The morphology of maize seedlings before and after drought treatment. (A) and (B) represents the maize seedlings at the early seedling stage with normal growth and drought treatment, respectively. The red arrow indicates the site of the root taking. ME represents mesocotyl, SR represents seminal root, LR represents lateral root, and PR represents primary root. (C), (D), and (E) represents the morphology of maize seedlings at the three-leaf stage treated for 6, 24 and 48 h, respectively. R represents roots. I, II and III represents control, 10% PEG-6000 and 25% PEG-6000 treated maize seedlings, respectively. (F) represents morphology of maize seedlings after 48 h of PEG-6000 treatment. (G), (H) and (I) represents primary root tip enlargement after 48 h of treatment with 0, 10%, and 25% PEG-6000, respectively. Fig. S2: Amino acid sequences alignment of ZmFARs using MegAlin. indicates the comparison of 23 proteins (except ZmFAR01), and (B) indicates the comparison of the all 24 ZmFARs. N-terminal WRKY-GCM1 zinc finger domain with the conserved CCHH amino acids, a central putative core transposase domain with the conserved DDG amino acids, and C-terminal SWIM zinc-finger domain of a CxCxnCXH motif are indicated, respectively. Black triangles represent conserved amino acids and red triangles represent amino acids not conserved at the site. Fig. S3: Synteny analysis of ZmFARs in maize genome. The collinearity relationship between different gene pairs was performed using MCScanX. Finally, the results were visualized using the the Dual Systeny Plot for MCScanX package in the TBtools. Fig. S4: The collinearity analysis of the ZmFAR family members between zea mays and four dicots, including Arabidopsis thaliana, Solanum tuberosum, Solanum lycopersicum, and Glycine max. Gray lines represent the collinear blocks between two plants in their genome. The MCScanX program was used to determine the covariance between different gene pairs and the results were visualized using [file peerj-12-17684-s001.zip › Supplemental materials/Supplemental figures/Fig. S2 Amino acid sequences alignment of ZmFARs using MegAlin..png]

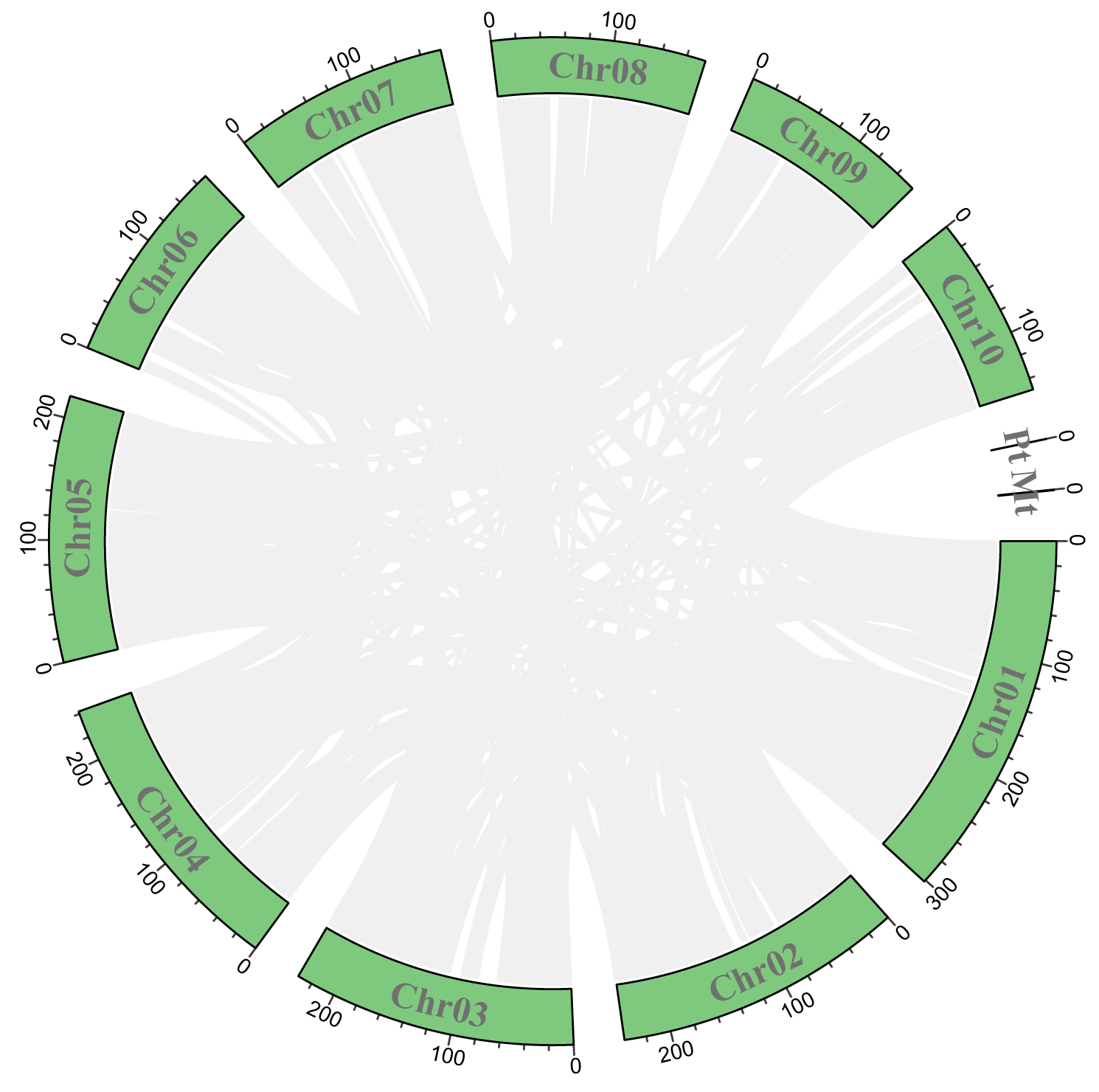

Supplement: Supplemental Information 1 — Fig. S1: The morphology of maize seedlings before and after drought treatment. (A) and (B) represents the maize seedlings at the early seedling stage with normal growth and drought treatment, respectively. The red arrow indicates the site of the root taking. ME represents mesocotyl, SR represents seminal root, LR represents lateral root, and PR represents primary root. (C), (D), and (E) represents the morphology of maize seedlings at the three-leaf stage treated for 6, 24 and 48 h, respectively. R represents roots. I, II and III represents control, 10% PEG-6000 and 25% PEG-6000 treated maize seedlings, respectively. (F) represents morphology of maize seedlings after 48 h of PEG-6000 treatment. (G), (H) and (I) represents primary root tip enlargement after 48 h of treatment with 0, 10%, and 25% PEG-6000, respectively. Fig. S2: Amino acid sequences alignment of ZmFARs using MegAlin. indicates the comparison of 23 proteins (except ZmFAR01), and (B) indicates the comparison of the all 24 ZmFARs. N-terminal WRKY-GCM1 zinc finger domain with the conserved CCHH amino acids, a central putative core transposase domain with the conserved DDG amino acids, and C-terminal SWIM zinc-finger domain of a CxCxnCXH motif are indicated, respectively. Black triangles represent conserved amino acids and red triangles represent amino acids not conserved at the site. Fig. S3: Synteny analysis of ZmFARs in maize genome. The collinearity relationship between different gene pairs was performed using MCScanX. Finally, the results were visualized using the the Dual Systeny Plot for MCScanX package in the TBtools. Fig. S4: The collinearity analysis of the ZmFAR family members between zea mays and four dicots, including Arabidopsis thaliana, Solanum tuberosum, Solanum lycopersicum, and Glycine max. Gray lines represent the collinear blocks between two plants in their genome. The MCScanX program was used to determine the covariance between different gene pairs and the results were visualized using [file peerj-12-17684-s001.zip › Supplemental materials/Supplemental figures/Fig. S3 Synteny analysis of ZmFARs in maize genome..png]

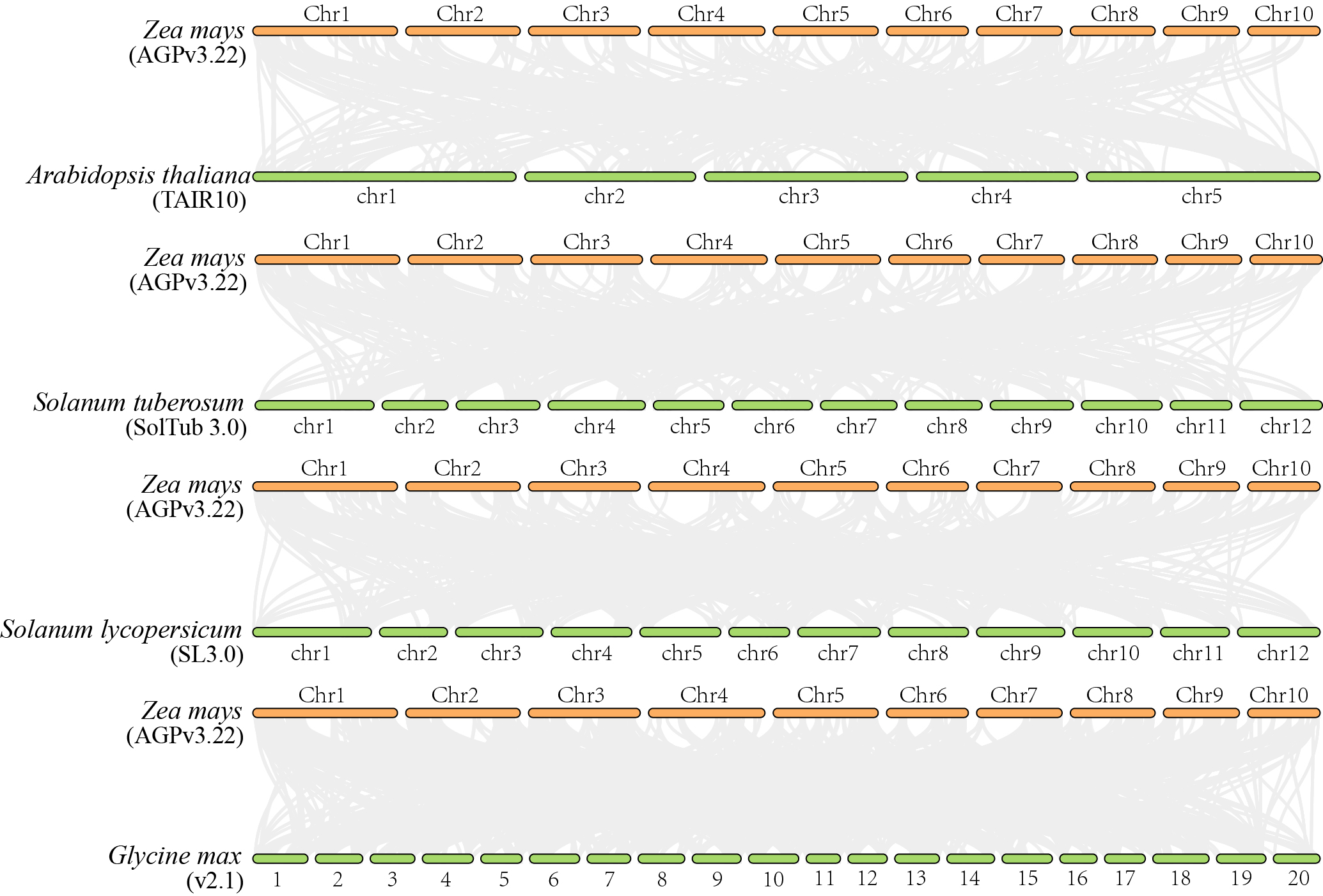

Supplement: Supplemental Information 1 — Fig. S1: The morphology of maize seedlings before and after drought treatment. (A) and (B) represents the maize seedlings at the early seedling stage with normal growth and drought treatment, respectively. The red arrow indicates the site of the root taking. ME represents mesocotyl, SR represents seminal root, LR represents lateral root, and PR represents primary root. (C), (D), and (E) represents the morphology of maize seedlings at the three-leaf stage treated for 6, 24 and 48 h, respectively. R represents roots. I, II and III represents control, 10% PEG-6000 and 25% PEG-6000 treated maize seedlings, respectively. (F) represents morphology of maize seedlings after 48 h of PEG-6000 treatment. (G), (H) and (I) represents primary root tip enlargement after 48 h of treatment with 0, 10%, and 25% PEG-6000, respectively. Fig. S2: Amino acid sequences alignment of ZmFARs using MegAlin. indicates the comparison of 23 proteins (except ZmFAR01), and (B) indicates the comparison of the all 24 ZmFARs. N-terminal WRKY-GCM1 zinc finger domain with the conserved CCHH amino acids, a central putative core transposase domain with the conserved DDG amino acids, and C-terminal SWIM zinc-finger domain of a CxCxnCXH motif are indicated, respectively. Black triangles represent conserved amino acids and red triangles represent amino acids not conserved at the site. Fig. S3: Synteny analysis of ZmFARs in maize genome. The collinearity relationship between different gene pairs was performed using MCScanX. Finally, the results were visualized using the the Dual Systeny Plot for MCScanX package in the TBtools. Fig. S4: The collinearity analysis of the ZmFAR family members between zea mays and four dicots, including Arabidopsis thaliana, Solanum tuberosum, Solanum lycopersicum, and Glycine max. Gray lines represent the collinear blocks between two plants in their genome. The MCScanX program was used to determine the covariance between different gene pairs and the results were visualized using [file peerj-12-17684-s001.zip › Supplemental materials/Supplemental figures/Fig. S4 The collinearity analysis of the ZmFAR family members between zea mays and four dicots, including Arabidopsis thalia.jpg]

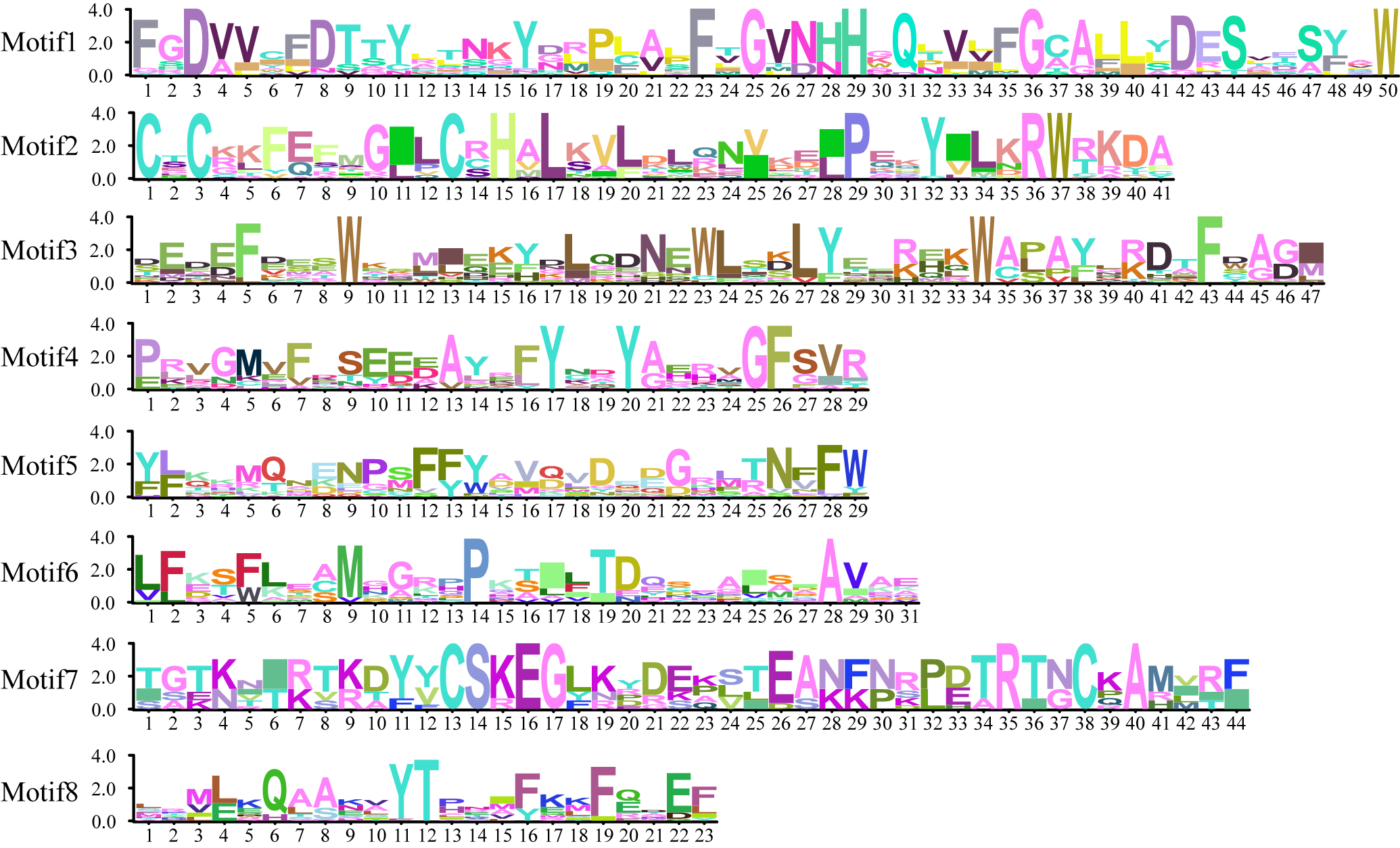

Supplement: Supplemental Information 1 — Fig. S1: The morphology of maize seedlings before and after drought treatment. (A) and (B) represents the maize seedlings at the early seedling stage with normal growth and drought treatment, respectively. The red arrow indicates the site of the root taking. ME represents mesocotyl, SR represents seminal root, LR represents lateral root, and PR represents primary root. (C), (D), and (E) represents the morphology of maize seedlings at the three-leaf stage treated for 6, 24 and 48 h, respectively. R represents roots. I, II and III represents control, 10% PEG-6000 and 25% PEG-6000 treated maize seedlings, respectively. (F) represents morphology of maize seedlings after 48 h of PEG-6000 treatment. (G), (H) and (I) represents primary root tip enlargement after 48 h of treatment with 0, 10%, and 25% PEG-6000, respectively. Fig. S2: Amino acid sequences alignment of ZmFARs using MegAlin. indicates the comparison of 23 proteins (except ZmFAR01), and (B) indicates the comparison of the all 24 ZmFARs. N-terminal WRKY-GCM1 zinc finger domain with the conserved CCHH amino acids, a central putative core transposase domain with the conserved DDG amino acids, and C-terminal SWIM zinc-finger domain of a CxCxnCXH motif are indicated, respectively. Black triangles represent conserved amino acids and red triangles represent amino acids not conserved at the site. Fig. S3: Synteny analysis of ZmFARs in maize genome. The collinearity relationship between different gene pairs was performed using MCScanX. Finally, the results were visualized using the the Dual Systeny Plot for MCScanX package in the TBtools. Fig. S4: The collinearity analysis of the ZmFAR family members between zea mays and four dicots, including Arabidopsis thaliana, Solanum tuberosum, Solanum lycopersicum, and Glycine max. Gray lines represent the collinear blocks between two plants in their genome. The MCScanX program was used to determine the covariance between different gene pairs and the results were visualized using [file peerj-12-17684-s001.zip › Supplemental materials/Supplemental figures/Fig. S5 Motif sequences identified for ZmFARs proteins..png]
